# Downregulation of rRNA Transcription Triggers Cell Differentiation

**DOI:** 10.1371/journal.pone.0098586

**Published:** 2014-05-30

**Authors:** Yuki Hayashi, Takao Kuroda, Hiroyuki Kishimoto, Changshan Wang, Atsushi Iwama, Keiji Kimura

**Affiliations:** 1 Graduate School of Life and Environmental Sciences, University of Tsukuba, Tsukuba Science City, Ibaraki, Japan; 2 Center for Tsukuba Advanced Research Alliance, University of Tsukuba, Tsukuba Science City, Ibaraki, Japan; 3 Department of Cellular and Molecular Medicine, Graduate School of Medicine, Chiba University, Chiba, Japan; Virginia Commonwealth University, United States of America

## Abstract

Responding to various stimuli is indispensable for the maintenance of homeostasis. The downregulation of ribosomal RNA (rRNA) transcription is one of the mechanisms involved in the response to stimuli by various cellular processes, such as cell cycle arrest and apoptosis. Cell differentiation is caused by intra- and extracellular stimuli and is associated with the downregulation of rRNA transcription as well as reduced cell growth. The downregulation of rRNA transcription during differentiation is considered to contribute to reduced cell growth. However, the downregulation of rRNA transcription can induce various cellular processes; therefore, it may positively regulate cell differentiation. To test this possibility, we specifically downregulated rRNA transcription using actinomycin D or a siRNA for Pol I-specific transcription factor IA (TIF-IA) in HL-60 and THP-1 cells, both of which have differentiation potential. The inhibition of rRNA transcription induced cell differentiation in both cell lines, which was demonstrated by the expression of the common differentiation marker CD11b. Furthermore, TIF-IA knockdown in an ex vivo culture of mouse hematopoietic stem cells increased the percentage of myeloid cells and reduced the percentage of immature cells. We also evaluated whether differentiation was induced via the inhibition of cell cycle progression because rRNA transcription is tightly coupled to cell growth. We found that cell cycle arrest without affecting rRNA transcription did not induce differentiation. To the best of our knowledge, our results demonstrate the first time that the downregulation of rRNA levels could be a trigger for the induction of differentiation in mammalian cells. Furthermore, this phenomenon was not simply a reflection of cell cycle arrest. Our results provide a novel insight into the relationship between rRNA transcription and cell differentiation.

## Introduction

The nucleolus is a major component of the nucleus and it is the site of ribosome biogenesis. The processes involved in ribosome generation require the transcription of ribosomal DNA (rDNA) genes by RNA polymerase I (Pol I). The initially transcribed ribosomal RNA (rRNA) is 47S rRNA, i.e., the so-called pre-rRNA, which is cleaved to form the mature 28S, 18S, and 5.8S rRNAs. Finally, the mature rRNAs are assembled with ribosomal proteins to generate functional ribosomes [1].

During these steps, the rate of rRNA transcription by Pol I is a major control point for ribosome biogenesis [2]. rRNA transcription requires the synergistic actions of two DNA-binding factors, the upstream binding factor (UBF) and the promoter selectivity factor (SL1/TIF-IB), both of which are essential for the recognition of a rDNA promoter by Pol I. UBF and SL1/TIF-IB interact with transcription initiation factor IA (TIF-IA), which mediates rRNA transcription by Pol I. The activity of TIF-IA is regulated by phosphorylation and it modulates the rate of rRNA transcription [3].

The regulation of rRNA transcription is physiologically important because the rate of rRNA transcription is coupled tightly to ribosome biogenesis, which subsequently determines the capacity of cells to grow and proliferate. For example, actively proliferating cells such as cancer cells require continuous rRNA transcription to ensure that their progeny cells have the capacity to support protein synthesis. In contrast, rRNA transcription is suppressed at low levels in slowly proliferating or arrested cells [3].

The downregulation of rRNA transcription is a mechanism that is involved in the response to various types of stress [4], [5], and it induces various processes, such as cell cycle arrest, apoptosis, or autophagy [6]–[9]. These processes are induced by p53 activation, which is mediated by two mechanisms: inhibition of HDM2, which is a ubiquitin ligase of p53, and the regulation of p53 modifications.

The first mechanism is mediated by nucleolar proteins, including nucleolin [10]; nucleophosmin [11]; nucleostemin [12]; ARF [13]; and ribosomal proteins, such as RPL5 [14], RPL11 [15], RPL23 [16], and RPS7 [17]. These proteins interact and inhibit HDM2, thereby resulting in p53 accumulation. The second mechanism is facilitated by MYB-binding protein 1a (MYBBP1A), which is localized in the nucleolus under normal conditions and it is translocated from the nucleolus to the nucleoplasm in response to DNA damage, thereby resulting in the acetylation and activation of p53 [6]. Thus, the downregulation of rRNA transcription affects cell growth and other cellular processes.

rRNA transcription is downregulated during differentiation (e.g., during myogenesis, osteogenesis, adipogenesis, granulopoiesis, and monocytic differentiation) [18]–[23], where cells transit from an actively proliferating state to a slowly proliferating or a cell cycle-arrested state, which is concomitant with the expression of lineage-specific transcription factors, e.g., MyoD and myogenin during myogenesis, Runx2 during osteogenesis, and C/EBP-β, -δ and -α during adipogenesis. The expression of lineage-specific factors increases with decreasing rRNA transcription because lineage-specific factors reduce rRNA transcription by occupying sites on rDNA promoters and interacting with UBF to suppress rRNA transcription [18].

The expression of c-Myc, a proto-oncogenic protein, is decreased in response to the transition from a proliferating state to a non-proliferating state during differentiation [19]. The downregulation of c-Myc decreases the expression of the Pol I-related transcription factors UBF, SL1/TIF-IB complex factors, and TIF-IA, thereby leading to the downregulation of rRNA transcription [20]. Thus, the downregulation of rRNA transcription is generally considered to be a consequence of differentiation. However, a recent report suggests that the downregulation of rRNA transcription promotes the early steps of differentiation in *Drosophila* germline stem cells [24].

In the present study, we used HL-60 and THP-1 cells and mouse hematopoietic stem cells (HSCs) in an ex vivo system to demonstrate that the downregulation of rRNA levels positively induces cell differentiation. Both cell lines are models of hematopoietic differentiation, which is one of the most widely investigated systems used to analyze cell differentiation. We specifically suppressed rRNA transcription using actinomycin D (Act D) or a siRNA specific for TIF-IA to examine whether cell differentiation was induced by the downregulation of rRNA levels.

We found that these treatments induced the expression of the differentiation marker CD11b, indicating that the downregulation of rRNA levels induced the differentiation of HL-60 and THP-1 cells. Using an ex vivo system of mouse HSCs, we then tested whether differentiation was induced by downregulating rRNA levels in normal hematopoietic cells. We found that TIF-IA knockdown (KD) decreased the proportions of immature cells but increased those of differentiated myeloid cells. Overall, these results indicate that the downregulation of rRNA transcription triggers cell differentiation.

## Results

### Act D inhibits rRNA transcription and induces the differentiation of HL-60 and THP-1 cells

The rRNA transcription levels are downregulated during differentiation [18]-[23]. The downregulation of rRNA transcription is generally considered to be a consequence of differentiation. Considering that the downregulation of rRNA transcription is involved in various cellular processes [6]–[9], it is also possible that downregulated rRNA levels induce differentiation. To test this possibility, we investigated whether the downregulation of rRNA levels induced differentiation using HL-60 and THP-1 cells. HL-60 cells are human promyelocytic leukemia cells and their granulocytic differentiation is induced by treatment with all-trans-retinoic acid (ATRA). THP-1 cells are human monocytic leukemia cells and their macrophage-like differentiation is induced by treatment with phorbol 12-myristate 13-acetate (PMA).

**Figure 1:**
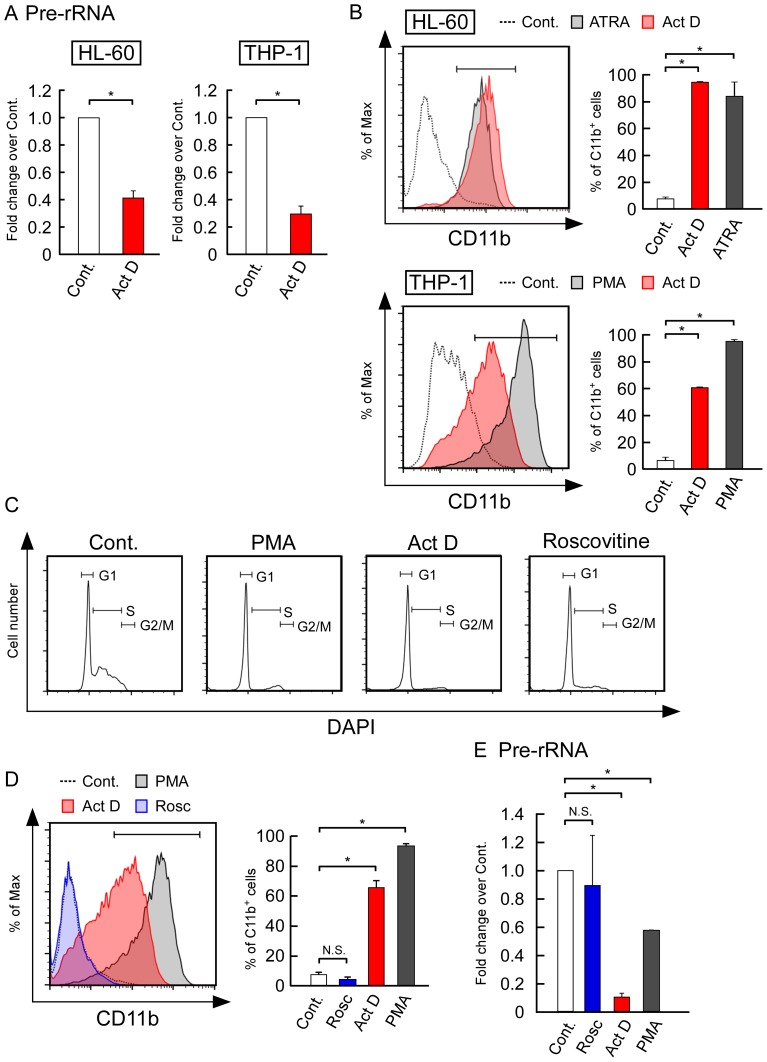
Suppression of rRNA transcription by actinomycin D (Act D) induced the differentiation of HL-60 and THP-1 cells. (A) A low concentration of Act D inhibited rRNA transcription in HL-60 and THP-1 cells. HL-60 and THP-1 cells were treated with 5 nM Act D for 24 h. The levels of pre-rRNA were determined by real-time quantitative PCR (RT-qPCR) and normalized by cell number. (B) Act D induced the expression of CD11b in HL-60 and THP-1 cells. Cells were cultured in the absence (control) or presence of all-trans-retinoic acid (ATRA) (1 µM), PMA (10 ng/mL), or Act D (5 nM) at 37°C. After 3 days, the CD11b expression levels were determined by flow cytometry (left panels). The corresponding mean percentages of CD11b-positive cells are shown in the left panels (right panels). (C, D) Inhibition of the cell cycle did not affect CD11b expression. THP-1 cells were treated with PMA (10 ng/mL), Act D (5 nM), or roscovitine (15 µM) for 3 days. (C) The DNA content was determined by DAPI and analyzed by flow cytometry. Similar results were obtained in three independent experiments. (D) The CD 11b expression levels were determined by flow cytometry. The corresponding mean percentages of CD11b-positive cells are shown in the left panels (right panels). (E) Roscovitine treatment did not affect the pre-rRNA levels. THP-1 cells were treated with PMA (10 ng/mL), Act D (5 nM), or roscovitine (15 µM) for 3 days. The pre-rRNA levels were determined by RT-qPCR and normalized by cell number. Values are expressed as the mean ± S.D., *n* = 3. *P<0.05. N.S.: *P*>0.05.

We treated these cells with Act D, which specifically inhibits rRNA transcription when used at a low concentration [25], [26]. Real-time quantitative PCR (RT-qPCR) analyses showed that the levels of pre-rRNA decreased after the Act D treatment in both of these cell types (Fig. 1A). Next, we used flow cytometry to analyze the expression levels of CD11b, a major differentiation marker for HL-60 and THP-1 cells. CD11b-positive cells increased after treatment with ATRA and PMA for 3 days, both of which were used as positive controls (Fig. 1B). The Act D treatment also induced CD11b expression in both of these cell types (Fig. 1B, left). The graphs on the right in Fig. 1B show that CD11b-positive cells increased significantly with the Act D treatment in both of these cell types. The Act D treatment also increased CD11c expression, which is another differentiation marker of THP-1 cells (Fig. S1A).

In contrast to the inhibition of rRNA transcription by a low concentration of Act D, the inhibition of pol II and pol III by α-amanitin did not induce CD11b expression in THP-1 cells (Fig. S2A). Furthermore, a higher concentration of Act D that inhibited all types of RNA polymerases did not effectively induce CD11b expression in THP-1 cells (Fig. S2B). Thus, the inhibition of pol II and pol III activity was not related directly to cell differentiation. These results suggest that the downregulation of rRNA levels by Act D induces the differentiation of HL-60 and THP-1 cells.

It is well known that rRNA transcription levels are tightly coupled to cell growth and cell cycle progression. The rRNA levels are suppressed when cell growth is limited [3], whereas the downregulation of rRNA transcription induces cell cycle arrest [6]. Decreased rRNA transcription levels and reduced cell growth are also observed during differentiation (Fig. S1B) [18]–[23]. Based on these observations, it is possible that differentiation is induced indirectly by cell cycle arrest after the Act D treatment, which suppresses rRNA transcription. To test this possibility, we treated THP-1 cells with roscovitine, a CDK inhibitor that inhibits cell cycle progression, and we analyzed the cell cycle profiles by flow cytometry. Most cells were arrested at the G1 phase after treatment with roscovitine, PMA, and Act D (Fig. 1C). However, roscovitine treatment did not induce the differentiation of THP-1 cells (Fig. 1D), which contrasted with our observations with Act D or PMA treatments (Fig. 1D). These results suggest that cell differentiation was not induced by cell cycle arrest.

Next, we tested the pre-rRNA levels by RT-qPCR to determine whether the pre-rRNA levels decreased after the roscovitine treatment. The pre-rRNA levels significantly decreased after treatment with PMA and Act D, both of which induced CD11b expression. However, the pre-rRNA levels were not affected by roscovitine, which also did not affect CD11b expression (Fig. 1E). Overall, these findings suggest that the downregulation of rRNA levels is essential to induce cell differentiation.

### TIF-IA KD induces the differentiation of HL-60 cells

**Figure 2:**
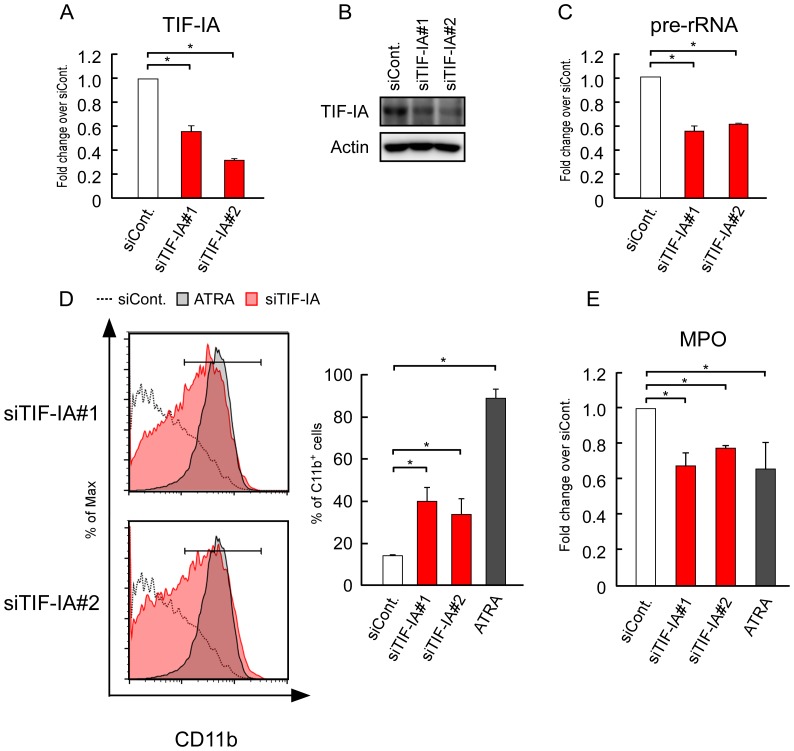
Suppression of rRNA transcription by TIF-IA KD induced the differentiation of HL-60. (A, B) siRNA-TIF-IA reduced the mRNA and protein levels of TIF-IA. HL-60 cells were transfected with siRNAs for luciferase (siCont.) and TIF-IA (siTIF-IA#1 or siTIF-IA#2), and cultured for 3 days. (A) The mRNA levels of TIF-IA were determined by RT-qPCR. (B) The protein levels of TIF-IA were determined by immunoblotting. (C) The pre-rRNA levels were determined by RT-qPCR. (D, E) TIF-IA KD induced the differentiation of HL-60 cells. (D) CD11b expression was determined by flow cytometry. ATRA (1 µM) was used as the positive control. The corresponding mean percentages of CD11b-positive cells are shown in the left panels (right panels). We present the same histograms for siCont. and ATRA in the upper and lower panels because these experiments were performed at the same time. (E) The MPO levels were determined by RT-qPCR and normalized by the cyclophilin levels. Values are expressed as the mean ± S.D., *n* = 3 (A, B, D). Values are expressed as the mean ± S.D., *n* = 4 (C). *P<0.05.

To further investigate the relationship between the rRNA transcription levels and differentiation, we treated HL-60 cells with a siRNA specific for TIF-IA, which is essential for Pol I-dependent transcription. TIF-IA KD decreases the rRNA transcription levels in MCF-7 and HeLa cells [6], [27]. The TIF-IA mRNA and protein levels were decreased in HL-60 cells after siRNA treatment (Fig. 2A, 2B). The pre-rRNA levels decreased in TIF-IA KD cells (Fig. 2C).

Next, we used flow cytometry to analyze CD11b expression in HL-60 cells at 3 days after TIF-IA KD, which showed that the CD11b-positive cells increased to moderate levels in TIF-IA KD cells compared with that in the control siRNA-treated cells (Fig. 2D). We then assessed the expression of myeloperoxidase (MPO), another differentiation marker of HL-60 cells. MPO expression decreases during the differentiation of HL-60 cells [28]. The RT-qPCR results showed that the MPO expression levels decreased significantly in TIF-IA KD cells to the same levels found in ATRA-treated cells (Fig. 2E).

Overall, these results indicate that the downregulation of rRNA levels induces the differentiation of HL-60.

### rRNA transcription levels decrease markedly from progenitor cells to differentiated cells in the mouse hematopoietic system

Our results indicated that the downregulation of rRNA levels promoted the differentiation of leukemia cell lines (Figs. 1 and 2). Next, we determined whether the downregulation of rRNA levels induces normal cell hematopoietic differentiation. To address this question, we used the mouse hematopoietic differentiation system, which is one of the most widely investigated in vivo differentiation systems.

**Figure 3:**
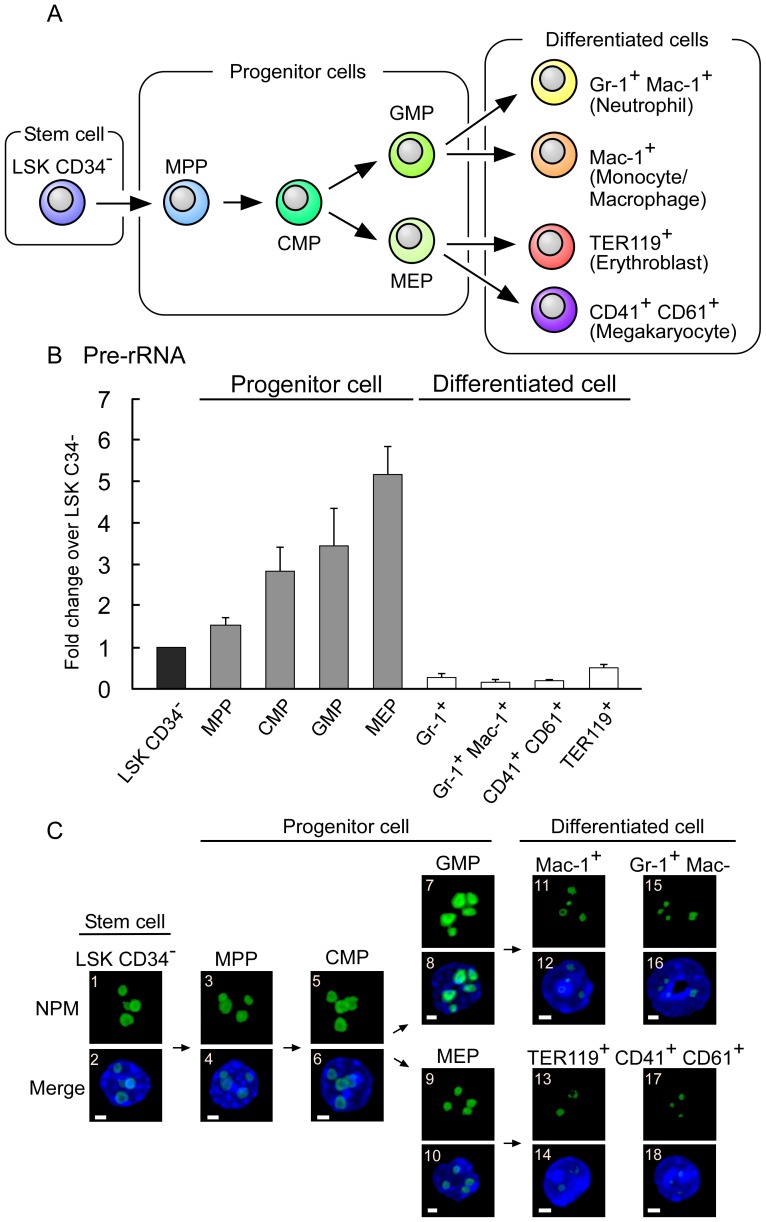
rRNA transcription levels decreased during mouse hematopoietic differentiation. (A) This scheme illustrates mouse hematopoietic differentiation into neutrophils, monocytes/macrophages, erythroblasts, or megakaryocyte cells. Nine types of cells were classified as stem cell, progenitor cells, and differentiated cells. The multipotent progenitor is denoted as MPP, the common myeloid progenitor as CMP, the granulocyte-monocyte progenitor as GMP, and the megakaryocyte-erythrocyte progenitor as MEP. (B) The pre-rRNA levels were reduced in the differentiated cells. The pre-rRNA levels of the nine types of hematopoietic cells were determined by RT-qPCR and normalized by cell number. (C) The nucleolar size was reduced in the differentiated cells. The nucleoli of the nine types of hematopoietic cells were immunostained using nucleophosmin (NPM) antibodies (panels with odd numbers). Merged images with DAPI are also shown (NPM, green; DAPI, blue; panels with even numbers). The white bar represents 2 µm. Values are expressed as the mean ± S.D., *n* = 5.

First, we compared the pre-rRNA levels in the various stages of mouse hematopoietic cells. We isolated mouse bone marrow and purified nine types of hematopoietic cells, including a stem cell, four types of progenitor cells, and four types of differentiated cells (Fig. 3A). To the best of our knowledge, for the first time, we showed that the pre-rRNA levels decreased dramatically during differentiation from progenitor cells to differentiated cells in the mouse hematopoietic system (Fig. 3B).

Next, we determined the sizes of the nucleoli because nucleolar size is correlated with rRNA transcription levels [8]. To examine the nucleolar size, we immunostained the nucleoli of mouse hematopoietic cells using anti-nucleophosmin antibodies. This showed that the differentiated cells contained much smaller nucleoli than the progenitor cells (Fig. 3C). These results demonstrated that the nucleolar size was reduced during differentiation from progenitor cells to differentiated cells.

### TIF-IA KD induces differentiation in an ex vivo mouse HSCs system

We found that rRNA transcription was downregulated during mouse hematopoietic differentiation (Fig. 3), which is similar to the regulation found in cell line models [18]–[23]. We also showed that the downregulation of rRNA levels induced the differentiation of HL-60 and THP-1 cells (Figs 1 and 2). Based on these results, we speculate that rRNA downregulation may also promote differentiation in normal hematopoietic cells.

**Figure 4:**
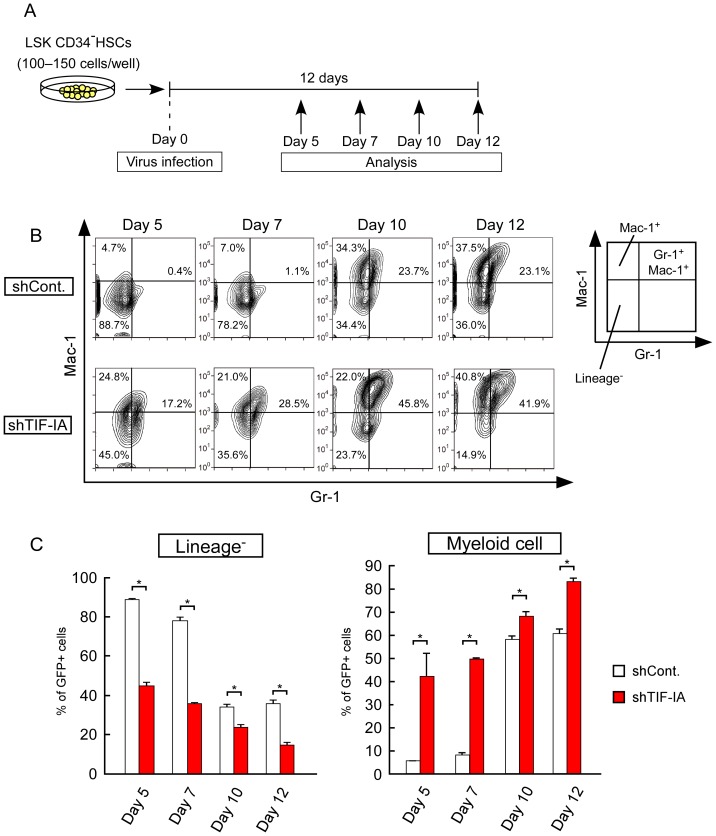
TIF-IA KD-induced cell differentiation of mouse HSCs in ex vivo culture. (A) Scheme showing the experimental procedure used for the HSC ex vivo culture system. The HSCs were purified from 8- to 12-week-old wild type mice. The purified HSCs were transduced with a lentivirus that expressed a shRNA against TIF-IA and cultured in media containing SCF and TPO. On days 5, 7, 10, and 12 after lentivirus transduction, myeloid differentiation was analyzed by flow cytometry. (B, C) TIF-IA KD promoted the myeloid differentiation of HSCs in culture. Anti-Mac-1 and anti-Gr-1 were used as myeloid cell markers. (B) The upper and lower panels show the results for the shControl and shTIF-IA cultures, respectively. Each panel shows the flow cytometric profiles of GFP^+^ transduced cells. (C) The percentages of lineage^−^ cells (Mac-1^−^Gr-1^−^) and myeloid cells (Mac1^+^ single positive and Mac-1^+^Gr-1^+^) among the GFP^+^ cells are shown as bar graphs. Values are expressed as the mean ± S.D., *n* = 3. *P<0.05.

We used an ex vivo culture system of HSCs to evaluate this hypothesis [29]. We purified mouse HSCs from mouse bone marrow and performed transduction using green fluorescent protein (GFP) with shRNA-luciferase (control) or GFP with shRNA-TIF-IA (TIF-IA KD). The transduced HSCs were cultured for 12 days in conditions that maintained their undifferentiated status. On days 5, 7, 10, and 12 after virus infection, we analyzed myeloid differentiation using specific antibodies (Gr-1 and Mac-1) and flow cytometry (Fig. 4A). The KD efficiency of shRNA-TIF-IA was determined by RT-qPCR in GFP-positive (GFP^+^) mouse hematopoietic cells on days 7 and 12 after virus infection and in RAW 264.7 cells (Fig. S3).

We monitored GFP expression as a marker of lentivirus-transduced cells. The panels in Fig. 4B and the percentages in Fig. 4C show the results after selecting GFP^+^ cells. As shown in Figs 4B and 4C, most of the cells were lineage^−^ cells (Gr-1^−^Mac-1^−^, immature cells) in the control population on day 5. However, the percentage of lineage^−^ cells decreased to approximately 50% in the TIF-IA KD population. In contrast to the lineage^−^ cells, the percentage of myeloid cells (Mac-1^+^ single positive and Mac-1^+^Gr-1^+^) increased in the TIF-IA KD population on day 5. In both the control and TIF-IA KD population, the lineage^−^ cells were decreased and the myeloid cells increased in a time-dependent manner. However, in the TIF-IA KD population, the myeloid cells were more enriched each day. These results suggest that TIF-IA KD accelerates myeloid differentiation in ex vivo cultures of HSCs.

Overall, our results suggest that the downregulation of rRNA transcription could be a trigger that induces differentiation.

## Discussion

The present study provides the first evidence that the downregulation of rRNA levels promotes the differentiation of mammalian cells. We speculate that two possible mechanisms may facilitate the induction of differentiation via downregulated rRNA levels: differentiation may be induced by reduced protein synthesis due to decreased ribosome biogenesis or differentiation may be induced by factors released from the nucleolus. We discuss these two possible mechanisms below.

### First possibility: Downregulation of rRNA transcription may induce differentiation via reduced protein synthesis

rRNA transcription is generally a rate-limiting step during ribosome biogenesis [2]. The downregulation of rRNA transcription decreases ribosome biogenesis, resulting in reduced protein synthesis [8]. Thus, the rRNA transcription levels are tightly coupled to protein synthesis. The polysome fraction, a complex of ribosomes and a single mRNA, decreases during the differentiation of HL-60 cells [30], which indicates that the overall protein synthesis is reduced during differentiation. However, we found that the expression levels of several proteins, such as the differentiation marker CD11b, increased during differentiation (Fig. 1B, 2D). Moreover, inhibiting protein synthesis using cycloheximide, puromycin, and emetine does not induce differentiation [31]. On the basis of these results, it can be concluded that overall protein synthesis is downregulated during differentiation, but the increased expression of several proteins is required. Therefore, reduced protein synthesis is a feature of differentiation, but it is not the overall mechanism that allows the downregulation of rRNA levels to induce differentiation.

### Second possibility: Downregulation of rRNA transcription may induce differentiation via factors released from the nucleolus

The nucleolus is a site of rRNA transcription, which comprises rDNAs, rRNAs, and nucleolar proteins. Transcribed rRNA, i.e., so-called pre-rRNA, is indispensable for maintaining the nucleolar structure as a scaffold of several nucleolar proteins [1], [32]. The downregulation of rRNA transcription causes the loss of this scaffold, resulting in the translocation of nucleolar proteins from the nucleolus to the nucleoplasm. Some of these translocated nucleolar proteins affect several cellular processes. For example, we previously reported that MYBBP1A is translocated to the nucleoplasm in response to DNA damage, which results in cell cycle arrest or apoptosis [6]. Nucleolar proteins, such as nucleophosmin, nucleostemin, and ARF, interact with HDM2 in the nucleoplasm and activate p53 [11]–[13].

In this study, we observed marked nucleolar shrinkage between transitions from progenitor cells to differentiated cells during mouse hematopoietic differentiation (Fig. 3C). This suggests that several nucleolar proteins are translocated to the nucleoplasm during differentiation.

The nucleolus is generally assumed to be a steady state structure and nucleolar proteins, such as UBF, nucleolin, and fibrillarin, are typically involved in ribosome biogenesis [3]. However, a recent high-throughput proteomic analysis showed that nucleolar proteins are exchanged dynamically with the nucleoplasm [33]. In 2005, a proteomic analysis detected over 700 human proteins in the nucleolus [33]. By 2008, the number of proteins identified had increased to over 4500 [34]. Some of these nucleolar proteins affect cell cycle arrest, apoptosis, autophagy, or stress responses in the nucleoplasm [6]–[9].

Some nucleolar proteins such as nucleolin and MYBBP1A interact with c-Myb, which is expressed in hematopoietic cells where it regulates their proliferation and differentiation [35], [36]. However, a possible relationship between the nucleolus and differentiation has not yet been elucidated. Based on previous reports and our own observations, we suggest the following hypothesis: the downregulation of rRNA transcription during differentiation affects the nucleolar morphology, which results in the translocation of nucleolar proteins to the nucleoplasm. In the nucleoplasm, these nucleolar proteins may regulate cell differentiation directly.

### Differentiation may be induced by various types of stress

The rRNA transcription levels are downregulated in response to intracellular and extracellular stress, such as ionizing irradiation (IR), hypoxia, heat shock, energy status, and chemical reagents, resulting in alterations of the nucleolar morphology [4], [37]. Stress-related nucleolar alterations induce the translocation of nucleolar proteins to the nucleoplasm, which results in cell cycle arrest or apoptosis [6]–[9]. This implies that the nucleolus is a central hub, which senses various types of stress via rRNA transcription levels and responds to stress through nucleolar proteins.

In stressed conditions, the homeostasis of an organism is maintained by the removal or repair of damaged cells as well as by replenishment with newly differentiated cells. Therefore, differentiation may be induced by stress to maintain homeostasis, as well as by cell cycle arrest or apoptosis. In fact, stress-related differentiation occurs in cell lines and mouse stem cells. For example, low-dose IR exposure to HL-60 cells induces their apoptosis and granulocytic differentiation [38]. Moderate hypoxic conditions induce the differentiation of human acute leukemic cell lines, including NB4, U937, and Kasumi-1 cells [39]. Genotoxic stress caused by IR results in the differentiation of melanocyte stem cells [40] and DNA damage responses cause the lymphoid differentiation of HSCs [41].

In the present study, we demonstrated that the downregulation of rRNA levels by the Act D treatment or TIF-IA KD induced the differentiation of leukemia cell lines and mouse hematopoietic cells (Figs 1, 2, and 4). Our experimental design mimicked cellular stress responses because the downregulation of rRNA transcription is caused by some types of stress. Thus, our findings suggest that the downregulation of rRNA levels connects these stress responses and cell differentiation. The nucleolus is a central hub for sensing stress, it can be speculated that the nucleolus mediates differentiation.

### Perspectives from this study

A recent study reported similar results during *Drosophila* germline stem cell differentiation [24]. Based on this report and our observations, the downregulation of rRNA transcription triggers cell differentiation and it is not simply a reflection of the cellular state during differentiation. Furthermore, this mechanism may be evolutionarily conserved. However, the mechanisms that link these phenomena remain unknown. We assume that nucleolar proteins are involved in this process, and we expect that we will find these proteins by screening for nucleolar proteins using siRNAs. Furthermore, although we obtained some novel insights into the differentiation process in vitro, the relationship remains unclear at the organism level. Finally, our future goals are to determine the mechanisms involved in this process and their biological significance in vivo.

## Materials and Methods

### Chemicals

Phorbol 12-myristate 13-acetate (PMA) and all-trans retinoic acid (ATRA) were purchased from Wako. Roscovitine (Rosc) were purchased from Santa Cruz.

### Cell culture

HL-60 human promyelocytic leukemia cells (HSRRB) and THP-1 human acute monocytic leukemia cells (Riken) were maintained in RPMI 1640 medium (Nacalai tesque) supplemented with 10% fetal bovine serum (FBS) (VERITAS). RAW264.7 Cells (Riken) were maintained in Dulbecco-modified Eagle medium (Sigma) medium supplemented with 10% fetal bovine serum (VERITAS), 100 units/mL penicillin, 100 units/mL streptomycin. All cells were maintained at 37°C in an atmosphere containing 5% CO_2_ and 100% humidity.

### siRNA transfection

For transfection of siRNAs, cells were transfected with 20 nM of siRNA using GenomONE-Si (ISHIHARA SANGYO KAISHA) according to the manufacturer's protocol. The sequence of siRNA duplexes is as follows: TIF-IA#1 (5′-CGACACCGUGGUUUCUCAUGCCAAU-3′), TIF-IA#2 (5′-AGGAUGUCUGCUAUGUAGAUGGUAA-3′). Stealth™ RNAi Luciferase reporter control duplex was used as a control.

### Real-time RT-qPCR

HL-60 and THP-1 cells were homogenized in 1 ml of sepasol (nacalai tesque) and total RNA was extracted according to the instruction manual. In RAW 264.7 cell and HSC ex vivo culture, we purified GFP+ cells using flow cytometry into TRIzol LS (Life Technologies) and extracted total RNA according to instruction manual. cDNA was synthesized from total RNA using RevatraAce reverse transcriptase (Toyobo) and random primers. Real-time PCRs were performed to amplify fragments representing for the indicated mRNA expression.

For analyzing pre-rRNA levels in mouse hematopoietic cells, cells were sorted into lysis solution of Cells Direct One-step qRT-PCR Kit (Invitrogen). According to the manufacture's protocol, real-time PCRs were performed to amplify fragments representing for pre-rRNA expression.

We used in the following primers: 5′-CCTAAAGCATACGGGTCCTG-3′ and 5′-TTTCACTTTGCCAAACACCA-3′ for human cyclophilin, 5′-CCATGAAAAAGGACATAGTG-3′ and 5′-CGTGTCAAAGGAGCTTGGT-3′ for human TIF-IA, 5′-GAACGGTGGTGTGTCGTTC-3′ and 5′-GCGTCTCGTCTCGTCTCACT-3′ for human pre-rRNA, 5′-TGCACACCCTCTTACTTCGG-3′ and 5′-GGTACTTCCTCATGGCCGTT-3′ for human MPO, 5′-TGACAGGGTGGTGACTTTACA-3′ and 5′-GCCATCCAGCCATTCAGTCTTG-3′ for mouse cyclophilin. 5′-GCCCTGGTTGAATAGAAGTCAG-3′ and 5′-CATGCTGAGACATGGTCTAAGG-3′ for mouse TIF-IA. 5′-TTTTTGGGGAGGTGGAGAGTC-3′ and 5′-CTGATACGGGCAGACACAGAAC-3′ for mouse pre-rRNA.

### Immunoblotting

For immunoblotting, HL-60 cells were harvested in 2 days of siRNA transfection. Cells were washed with PBS, and lysed in radioimmune precipitation assay buffer (25 mM Tris-HCl, pH 7.6, 150 mM NaCl, 1% Nonidet P-40, 1% Triton X-100, 1% sodium deoxy-cholate, and 0.1% SDS). Lysates were cleared by centrifugation at 16,100×g for 20 min at 4°C. Protein concentration was determined by BCA kit (Thermo Scientific). Extracted proteins were separated by SDS-PAGE, transferred onto PVDF membranes (Millipore). After blocking with 5% skim milk in TBS-T buffer (20 mM Tris at pH 7.5, 150 mM NaCl, and 0.05% Tween 20) for 30 min, the membranes were incubated with anti-TIF-IA (H-300, Santa Cruz) and anti-β-Actin (C4, Santa Cruz) antibodies for overnight at 4°C. After washing with TBS-T buffer, the membranes were incubated with horseradish peroxidase-conjugated secondary antibody for 1 h. Bands were detected with Immobilon Western blotting detection kit (Millipore).

### Flow cytometric analysis of CD11b and CD11c expression

HL-60 and THP-1 were washed with phosphate-buffered saline (PBS) containing 2% FBS (2% FBS/PBS). Allophycocyanin (APC)-conjugated anti-human CD11b monoclonal antibody (BioLegend) and FITC-conjugated anti-human CD11c monoclonal antibody (eBioscience) were added and incubated for 1 h in the dark on ice. After washing with 2% FBS/PBS, the cells were suspended in 2% FBS/PBS supplemented with 1 µg/ml of Propidium iodide (PI). CD11b and CD11c expression was measured on the FACS Aria II (BD Bioscience) and analyzed by Flowjo software.

### Cell cycle analysis

Cells were washed with 2% FBS/PBS and suspend in 4′,6-Diamidino-2-Phenylindole, Dihydrochloride (DAPI) staining solution (0.1% (v/v) Triton X-100 and 1 µg/mL DAPI in PBS). Suspended cells were kept in the dark on ice for 30 min. DAPI fluorescence was detected using UV light laser on FACS Aria II.

### MTT assay

For MTT assay, THP-1 cells were seeded by 5,000 cells per well in 96-well plate. MTT assay was performed using MTT Cell Count Kit (Nacalai Tesque). The kit used according to instruction manual.

### Lentivirus production

Lentivirus production is previously described [29]. Briefly, lentiviral vectors (CS-H1-shRNA-EF-1α-EGFP) expressing shRNA against murine *Rrn3* (target sequence: GGCACAGATCTCAAGATAC,) and *luciferase* were prepared. 293T cells were transfected with three plasmids: packaging construct (pCAG-HIVgp), VSV-G- and Rev-expressing construct (pCMV-VSV-G-RSV-Rev), and SIN vector construct (CS-H1-shRNA-EG). Supernatants from transfected cells were concentrated by centrifugation at 6,000×*g* for 16 h, and then resuspended in α-MEM supplemented with 1% FBS (1/100 of the initial volume of supernatant). Viral titers were determined by infection of Jurkat cells (a human T-cell line).

### Lentivirus transduction to RAW264.7 cells

RAW264.7 cells were transduced with concentrated lentiviral vector stocks at a multiplicity of infection (MOI) of 5 in the presence of 8 µg/ml Polybrene. The expression of green fluorescent protein (GFP) in the RAW264.7 cells was confirmed by flow cytometry, and cells expressed GFP were purified for RT-qPCR.

### Mice

C57BL/6J mice were purchased from Japan SLC (CLEA JAPAN). For every experiment using mice, 8-12-old-week male mice were used.

### Cell Sorting on mice hematopoietic cells

Mouse CD34^–^LSK HSCs (Lin^−^Sca-1^−^c-Kit^−^CD34^−^) were purified from bone marrow (BM) of three 8-12-week-old male mice. BM mononuclear cells were isolated on Ficoll-Paque PLUS (GE Healthcare) and incubated with a mixture of biotin-conjugated mouse antibodies against lineage markers (Lin) including anti-Gr-1, Mac-1, B220, CD4, CD8 and Ter-119 monoclonal antibodies (BD Pharmingen). The cells were further stained with FITC-conjugated anti-CD34, PE-Cy7-conjugated Sca-1, APC-conjugated anti-c-Kit (BD Pharmingen), and Alexa Fluor 700-conjugated anti-CD16/32 antibodies (eBioscience). For isolating mature cells, BM mononuclear cells were stained with Biotin-conjugated anti-Mac-1 and PE-conjugated anti-Gr-1, or FITC-conjugated anti-Ter-119, APC-conjugated anti-CD41, and Biotin-conjugated anti-CD61 antibodies. Biotinylated antibodies were detected with streptavidin-APC-Cy7 antibody (BD Pharmingen). Dead cells were eliminated by staining with PI (1 µg/ml). Analysis and sorting were performed on a FACS Aria II.

### In vitro culture of mouse HSCs

In vitro culture experiments for mouse HSCs was performed as described [29]. Briefly, CD34^−^LSK cells were deposited into recombinant fibronectin fragment (Takara Shuzo, Otsu, Japan)-coated 96-well micro-titer plates, at 100–150 cells per well, and incubated in α-MEM supplemented with 1% FBS, 100 ng/ml mouse stem cell factor (SCF) (Wako), and 100 ng/ml mouse thrombopoietin (TPO) (Wako) for 24 h. Then, cells were transduced with a lentivirus vector at a MOI of 1,500 for 24 h. After transduction, cells were further incubated in S-Clone SF-O3 (Sanko Junyaku, Tokyo, Japan) supplemented with 0.2% BSA, 50 ng/ml SCF, and 50 ng/ml TPO for 12 days. Medium change was performed every 3 day after transduction. On the day 5, 7, 10, and 12 after virus infection, myeloid differentiation was analyzed using APC conjugated anti-Mac-1 and PE conjugated anti-Gr-1 antibodies and flow cytometry.

### Single cell immunofluorescence

Single cell immunostaining was performed as described [42]. Hematopoietic cells were sorted into 2% FBS/PBS. The cells were centrifuged and suspended in S-Clone SF-O3 (EIDIA) and seeded poly-L-lysine coated 10 well slide glasses. Cells were incubated for 3 h at 37°C. After incubation, cells were fixed with 4% paraformaldehyde at room temperature for 30 min and permeabilized with 0.1%Triton X-100 buffer (20 mM HEPES, 150mM KCl, 0.5% Triton X-100) for 15 min at room temperature. Cells were blocked with 3% BSA for 30 min, and incubated with anti-NPM antibody at 4°C for overnight. Cells were washed with PBS and incubated with Alexa Fluor 594-conjugated secondary antibody (Invitrogen) at room temperature for 1 h. Cells were washed again with PBS and mounted on slides using Vectashield with DAPI (Vector Laboratories). Immunofluorescence was performed using LSM 700 (Carl Zeiss).

### Statistical analysis

All the data are representative of indicated different experiments. Statistical analysis was performed using t-test.

### Ethics statement

All experiments were performed in accordance with the Declaration of Helsinki and were approved by University of Tsukuba Ethics Committee for Animal Experiments.

## Supporting Information

Figure S1 **Act D treatment induced the differentiation of THP-1 cell's differentiation.** (A) THP-1 cells were treated with PMA (10 ng/mL) and Act D (5 nM) for 3 days or 7 days, The levels of CD11b and CD11c expression levels were determined by flow cytometry. (B) THP-1 cells were treated with PMA (10 ng/mL) and Act D (5 nM). After indicated time, MTT assays was were performed after the times indicated.
(TIF)

Figure S2 **Inhibition of pol II or pol III activity did not promote the differentiation of THP-1 cells.** (A) THP-1 cells were treated with PMA (10 ng/mL), Act D (5 nM), and α-amanitin (2 µg/mL) for 24 h. After 24 h, the medium was changed and the cells were cultured for 2 days. The CD11b expression levels were determined by flow cytometry. (B) THP-1 cells were treated with different concentrations of Act D (5, 50, or 500 nM) for 48 h. The CD11b expression levels were determined by flow cytometry.
(TIF)

Figure S3 **KD efficiencies of TIF-IA in RAW 246.7 cells and mouse hematopoietic cells.** (A) Purified mouse HSCs were infected with GFP-expressing lentiviruses that contained shLuc (shCont.) or shTIF-IA. GFP^+^ cells were collected by flow cytometry on the days indicated. The TIF-IA levels were determined by RT-qPCR and normalized by cyclophilin. (B) RAW246.7 cells were transduced with lentiviruses that contained the shRNAs indicated and cultured for 2 days. The TIF-IA levels were determined by RT-qPCR and normalized by the cyclophilin levels. Data are expressed as the mean ± S.D.
(TIF)
