# Supplementary figures and images for: Downregulation of rRNA Transcription Triggers Cell Differentiation
Source: PLoS One. 2014 May 30;9(5):e98586. doi: 10.1371/journal.pone.0098586 (PMC4039485; doi:10.1371/journal.pone.0098586)

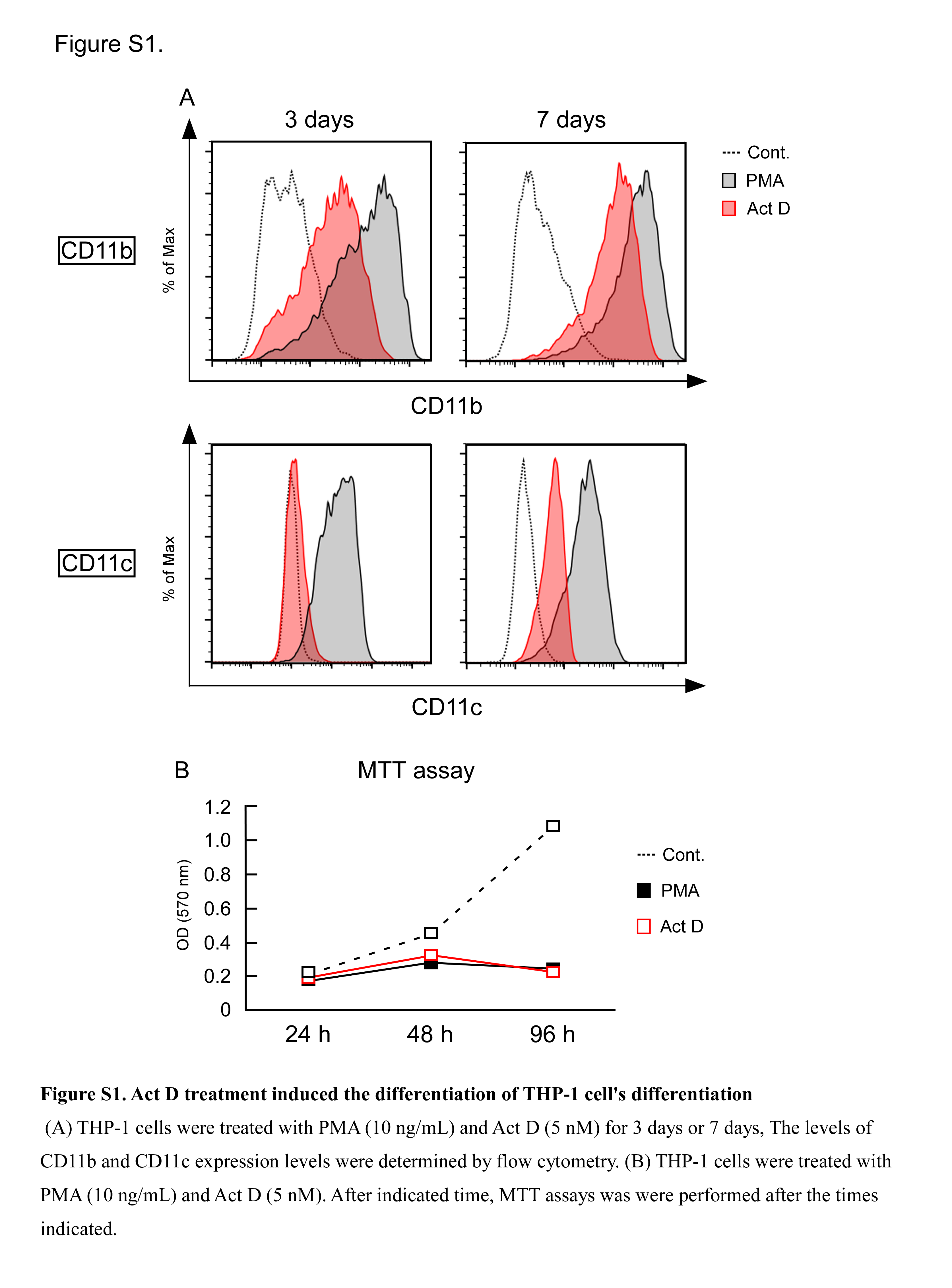

Supplement: Figure S1 — Act D treatment induced the differentiation of THP-1 cell's differentiation. (A) THP-1 cells were treated with PMA (10 ng/mL) and Act D (5 nM) for 3 days or 7 days, The levels of CD11b and CD11c expression levels were determined by flow cytometry. (B) THP-1 cells were treated with PMA (10 ng/mL) and Act D (5 nM). After indicated time, MTT assays was were performed after the times indicated. (TIF) [file pone.0098586.s001.tif]

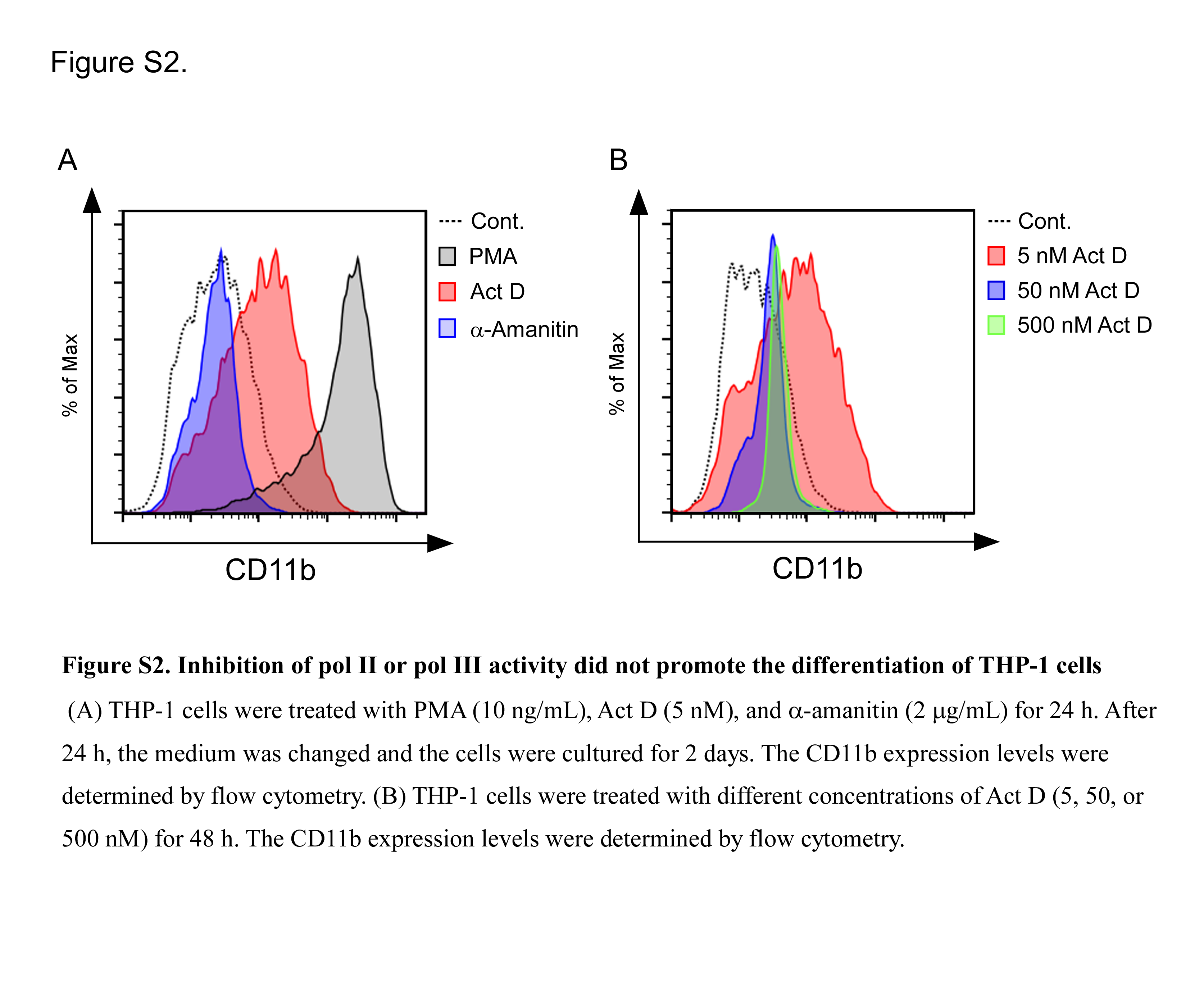

Supplement: Figure S2 — Inhibition of pol II or pol III activity did not promote the differentiation of THP-1 cells. (A) THP-1 cells were treated with PMA (10 ng/mL), Act D (5 nM), and α-amanitin (2 µg/mL) for 24 h. After 24 h, the medium was changed and the cells were cultured for 2 days. The CD11b expression levels were determined by flow cytometry. (B) THP-1 cells were treated with different concentrations of Act D (5, 50, or 500 nM) for 48 h. The CD11b expression levels were determined by flow cytometry. (TIF) [file pone.0098586.s002.tif]

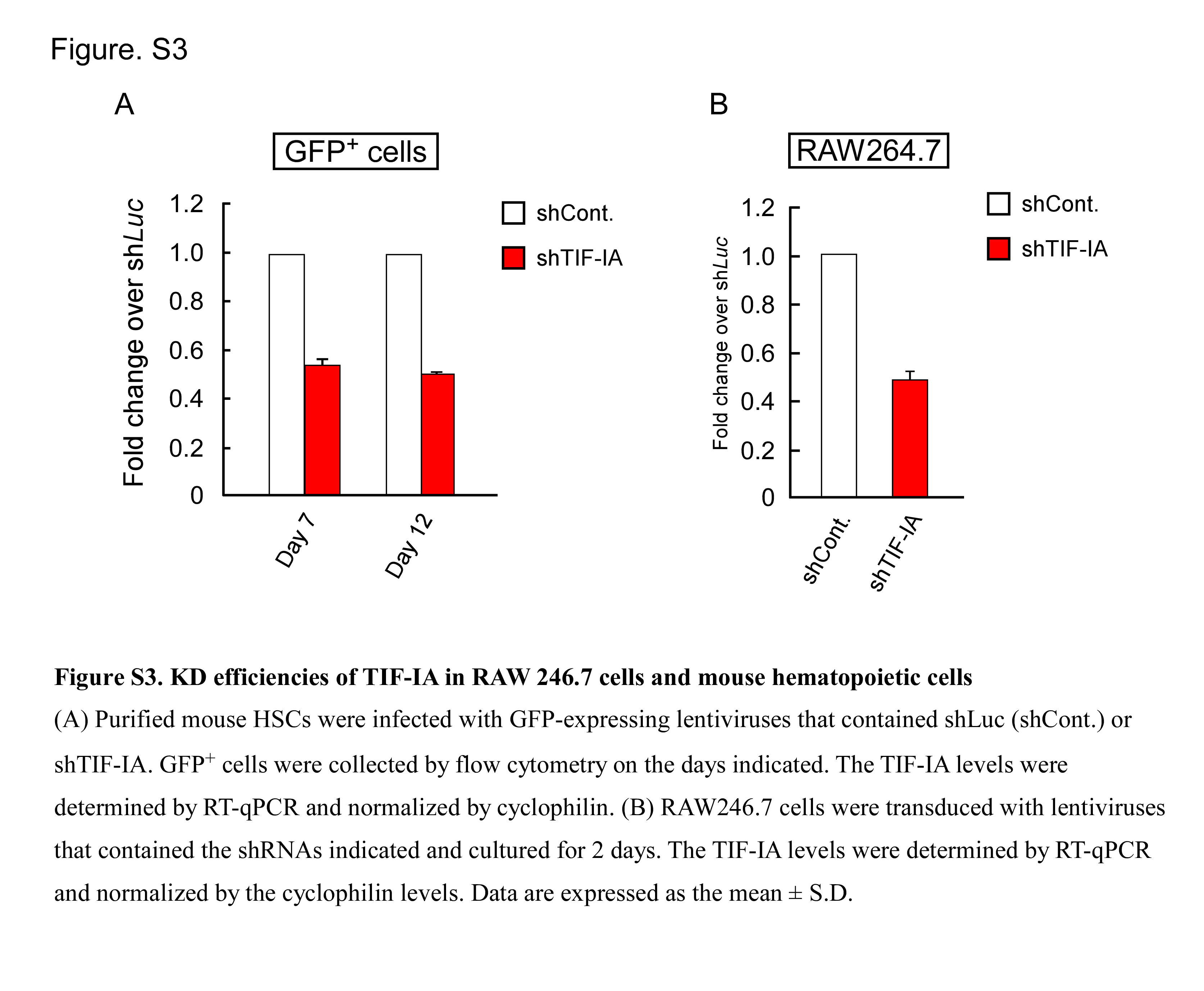

Supplement: Figure S3 — KD efficiencies of TIF-IA in RAW 246.7 cells and mouse hematopoietic cells. (A) Purified mouse HSCs were infected with GFP-expressing lentiviruses that contained shLuc (shCont.) or shTIF-IA. GFP+ cells were collected by flow cytometry on the days indicated. The TIF-IA levels were determined by RT-qPCR and normalized by cyclophilin. (B) RAW246.7 cells were transduced with lentiviruses that contained the shRNAs indicated and cultured for 2 days. The TIF-IA levels were determined by RT-qPCR and normalized by the cyclophilin levels. Data are expressed as the mean ± S.D. (TIF) [file pone.0098586.s003.tif]
